# Early life predictors of positive change during the coronavirus disease pandemic

**DOI:** 10.1186/s40359-021-00586-7

**Published:** 2021-05-18

**Authors:** Maria E. Bleil, Bradley M. Appelhans, Alexis S. Thomas, Steven E. Gregorich, Neal Marquez, Glenn I. Roisman, Cathryn Booth-LaForce, Kyle Crowder

**Affiliations:** 1 Department of Child, Family, and Population Health Nursing, University of Washington, Box 357262, Seattle, WA 98195 USA; 2 Department of Preventive Medicine, Rush University Medical Center, Chicago, IL USA; 3 Department of Medicine, University of California San Francisco, San Francisco, CA USA; 4 Department of Sociology, University of Washington, Seattle, WA USA; 5 Institute of Child Development, University of Minnesota, Minneapolis, MN USA

**Keywords:** Coronavirus disease pandemic, COVID-19, Early life adversity, Stress, Neighborhood disadvantage, Socioeconomic status, Resilience, Positive change

## Abstract

**Background:**

The COVID-19 pandemic is a crisis unprecedented in its size and scope. Yet studies of resilience suggest most individuals will successfully negotiate this challenge and some may even experience growth and positive change. Some evidence suggests that the capacity to enact positive change in the face of adversity may be shaped by early life experiences.

**Methods:**

In a subset of 374 participants (57% female, mean age = 29 years) in the Study of Early Child Care and Youth Development (SECCYD), a longitudinal, birth cohort, prospective models were tested to determine whether early life adversities in family and neighborhood contexts predict positive change events in response to the COVID-19 pandemic. Childhood family and neighborhood contexts were assessed using a combination of self-report questionnaires and US Census data. Adulthood positive change events (e.g., becoming more appreciative of things usually taken for granted) were assessed using the Epidemic-Pandemic Impacts Inventory (EPII).

**Results:**

In regression analyses, neighborhood disadvantage in childhood, measured both by objective and subjective assessments, predicted a higher number of positive change events in response to the COVID-19 pandemic (*β* = .18, *p* = .004 and *β* = .15, *p* = .006, respectively). Examination of the positive change event subscales showed neighborhood disadvantage in childhood predicted increases in events related to ‘perspective taking and charitable giving’ (*β* = .20, *p* = .022 and *β* = .17, *p* = .002, respectively) and improved ‘social relationships’ (*β* = .18, *p* = .004 and *β* = .13, *p* = .020, respectively), but not to positive ‘health behaviors’ (*ps* > .05). All associations were independent of sociodemographic factors and childhood family dysfunction.

**Conclusions:**

Findings suggest that neighborhood disadvantage in childhood may shape prosocial responses to stress in adulthood, potentially through early life adaptions to stress that are protective when facing adversity. There are several notable implications of the study findings. Although adversity in early life has clear negative impacts, it is possible that adversity experiences may also provide opportunities to develop adaptive strategies that foster resilience and growth when facing stress. Intervention efforts should consider leveraging such stress-adapted strengths to reduce the many negative impacts of early life adversity.

## Background

Since the first case of coronavirus disease 2019 (COVID-19; the disease caused by the SARS-CoV-2 virus) was diagnosed in the United States [1], Americans have been disproportionately affected by the SARS-CoV-2 virus. Only 4% of the world’s population, Americans represent more than 20% of the total number of deaths globally [2, 3]. In addition, prolonged periods of illness are typical in patients with severe COVID-19 and in a sizable proportion of those with mild symptoms [4, 5]. Taken together, the morbidity and mortality costs of the COVID-19 pandemic in the United States have been immense as have the many societal disruptions related to efforts to thwart its spread (e.g., school and business closings). As of September 2020, 60% of American children in the public school system began the school year attending classes remotely and 19.4 million Americans reported they were unable to work due to pandemic related workplace closures [6, 7]. Moreover, increases in psychological stress and family dysfunction over this period have resulted in increases in anxiety and depression, substance use, and suicidal ideation, as well as domestic violence and child maltreatment [8–10].

Despite the numerous negative impacts of the COVID-19 pandemic, research based on prior disasters and personal tragedies suggests that most individuals will successfully endure these challenges and some may even experience growth and positive change in their lives. Studies of post-traumatic stress disorder, for example, indicate that the majority of individuals exposed to severe stressors such as disasters (e.g., flooding, terrorist attacks) or military combat do not experience significant distress or are able to return to baseline levels of functioning and psychological well-being without intervention [11–13]. Studies of individual-level stressors replicate and extend these findings. That is, a significant proportion of individuals exposed to stressful life events, such as facing a diagnosis of cancer, not only maintain function but engage in a process termed “benefit-finding” or “stress-related growth”. This process describes positive life changes experienced as a result of adversity, potentially leading to better than expected psychological health and less distress long-term [14–16].

The capacity of individuals to respond, adapt, and even grow in response to stress may be rooted in early life contexts. Studies of early life stress show greater exposure to adversity in childhood predicts adulthood risk for poor mental and physical health outcomes and that the timing of such exposures may be more impactful when experienced in childhood [17]. Adversity exposures threaten a child’s emotional or physical security and are hypothesized to disrupt typical development by promoting alterations in physiological processes underpinning decrements in mental and physical health [17–20]. To date, this literature has been limited in its focus on parameters of risk in relation to negative outcomes with little consideration for ways in which responses to stress may be adaptive in facilitating positive outcomes or in advancing future responses to stressful life events. Alternatively, a recent model advanced by Ellis and colleagues [21] highlights the strengths (vs. deficits) of adversity-exposed children that may develop as adaptations to stress. Such adaptations or “hidden talents,” in turn, may serve to enhance stress-adapted skill sets that advantage these individuals in particular contexts or when encountering adversity in the future. In sum, although exposure to adversity in early life has clear negative impacts on adult well-being, it is plausible that adversity experiences may also provide opportunities to develop resilience and adaptive socioemotional and behavioral strategies that enable individuals to enact positive change when faced with stressful life events.

The current study included a subset of 374 participants in a follow-up study of the landmark NICHD Study of Early Child Care and Youth Development (SECCYD) who additionally completed a questionnaire inquiring about changes they experienced as a result of the COVID-19 pandemic. The primary study objective was to determine whether adversity experienced in early childhood might influence the capacity of individuals as adults to enact positive change in response to stress. Specifically, prospective models examined early life contexts related to family and neighborhood environments in childhood in relation to self-reported positive changes in adulthood, experienced in response to the COVID-19 pandemic—a stressful life event unprecedented in its size and scope. Measured changes pertained to increases in perspective-taking and charitable giving, as well as improvements in social relationships and health behaviors. Models accounted for relevant sociodemographic variables, including gender, race/ethnicity, and socioeconomic status indexed in childhood and adulthood as well as childhood family dysfunction. Separate models were fit considering childhood neighborhood environments assessed objectively, through use of US Census data, and subjectively, through retrospective reports of neighborhood conditions.

## Methods

### Participants

Participants were drawn from the NICHD Study of Early Child Care and Youth Development (SECCYD), a prospective investigation of children and their families recruited at birth to examine trajectories of health and development across early childhood (birth to 54 months), middle childhood (kindergarten through to fifth grade), and adolescence (sixth grade through 15 years of age). Families were recruited from 10 geographically diverse study sites in the United States: Charlottesville, VA; Irvine, CA; Lawrence, KS; Little Rock, AR; Madison, WI; Morganton, NC; Philadelphia, PA; Pittsburgh, PA; Seattle, WA; and Wellesley, MA. All mother-infant dyads of babies born within pre-selected 24-h intervals at participating hospitals were screened. Families were excluded if the: (1) mother was < 18 years old; (2) mother was non-English speaking; (3) family was re-locating within 1 year; (4) infant or mother had a serious medical problem; (5) mother had a substance use disorder; (6) infant was being given up for adoption; (7) family lived > 1 h away from the study site; (8) family was already participating in another study; and/or (9) family refused to participate in the initial study interview. Additional sampling requirements were employed (e.g., 10% recruitment of single-parent households) to ensure that the socio-demographic composition of the final sample (*N* = 1364 families) was similar to the population of families living in the same geographic regions, according to the 1990 US Census.

Currently, an SECCYD follow-up study—the Study of Health in Early and Adult Life (SHINE)—is underway to locate these children now in adulthood (late 20 s). SHINE entails an in-person study visit collecting extensive social, behavioral, and health data with the goal of examining effects of early life adversity exposures, and the mechanisms of these effects, on trajectories of health and disease risk over time. Retained for analysis in the current study were participants in SHINE between January 2018 and March 2020 who subsequently completed a self-report questionnaire assessing impacts of the COVID-19 pandemic. The questionnaire was administered over a 3-month period between June and August 2020, beginning approximately 4 months after the first case of COVID-2019 was diagnosed in the United States [1]. The newly collected questionnaire data were then merged with the relevant SECCYD and SHINE data for analysis. Of the 430 eligible SHINE participants, 374 (87.0%) completed the questionnaire. This subset of participants is being examined in the current study ahead of the completion of the SHINE Study due to its timely focus on understanding individual-level impacts of the COVID-19 pandemic and how these impacts may be shaped by early life factors. Comparison of the current subset of participants (*n* = 374) to all non-participants from the original SECCYD (*n* = 990) showed the current sample experienced higher SES in childhood, indexed by a higher mean number of years of parental education (*M*[*SD*] = 14.9[2.2] vs. 14.0[2.4]; *t*[1362] = 6.0, *p* = 0.000) and higher mean family income-to-needs ratio between child’s age 1 month and grade 1 (*M*[*SD*] = 3.6[2.3] vs. 3.3[2.8]; *t*[1353] = 2.0, *p* = 0.043). Informed consent for the original NICHD SECCYD was obtained from parents and assent was obtained from children when they were old enough to do so. Informed consent for the SECCYD follow-up study, SHINE, was obtained from the now adult children. For the SECCYD, the research was approved by the Institutional Review Boards (IRB) of each university-based study site (e.g., University of California, Irvine IRB; University of Virginia IRB Social and Behavioral Science; Temple University Human Research Protection Program). For SHINE, the research was approved by the Human Subjects Division of the University of Washington. All methods were carried out in accordance with relevant guidelines and regulations.

## Measures

### Sociodemographic Information

The sociodemographic variables included gender, race/ethnicity, child SES, and adult SES. *Child SES* was indexed by a composite of mother and father/partner’s education and family income-to-needs ratio. Educational attainment of the mother and father/partner at child’s age 1 month was ascertained by self-report: 1 = less than high school; 2 = high school or general education diploma; 3 = some college or vocational degree; 4 = college degree; 5 = some graduate school or master’s degree; and 6 = graduate degree greater than a master’s degree. Income-to-needs ratio (i.e., family income expressed as a proportion of the federal poverty line for a family of a particular size) was derived by self-report of family income at child’s ages 1, 6, 15, 24, 36, 54 months and in kindergarten and grade 1. Separate means, computed across the indicated time points for parental education and income-to-needs ratio, were then standardized, summed, and re-standardized to produce a single composite of SES in childhood. *Adult SES* was indexed by a composite of the participant’s individual-level education and household income at the time of the SECCYD follow-up study (SHINE). Educational attainment of the participant was ascertained by self-report according to the same categories described above regarding parental education. Total combined household income was ascertained by self-report in categories ranging between < $5,000 and $300,000 or more. Household income included income from all sources (e.g., wages, veteran’s benefits, help from relatives, and rent from properties) in the past 12 months, which was then divided by the number of dependents supported by the income. The indicated values were then standardized, summed, and re-standardized to produce a single composite of SES in adulthood.

### Predictors: early life factors

#### Child neighborhood disadvantage (objective assessment)

Residential addresses were documented and updated continuously as a part of families’ participation in the original NICHD SECCYD. Addresses were geocoded and a time series database was created describing neighborhood-level socioeconomic indicators for these locations over time. Neighborhood variables were derived from the 1990 and 2000 decennial US Censuses, including (1) neighborhood-level educational attainment indexed by the percent of residents (among persons 25 years and older) without a high school diploma or GED; (2) neighborhood-level income indexed by median family income; (3) neighborhood-level employment indexed by the percent of working-age male residents (among persons 25 years and older) who are either unemployed or out of the labor force; (4) neighborhood-level public assistance indexed by the percent of families receiving public assistance; and (5) neighborhood-level poverty indexed by the percent of families with income below the federal poverty threshold. Neighborhood variables were extracted at the tract level and older censuses were crosswalked to the 2010 US Census to account for changes in tract boundaries over time. In this way, variables reflect neighborhood-level socioeconomic indicators from the 1990 and 2000 decennial US Censuses standardized to 2010 US Census boundaries, allowing for comparison of variables over time. In addition, all dollar-valued variables (i.e., median family income) were adjusted to 2010 US dollars. Linear interpolation was used to estimate data for intercensal years. For missing locations, the last observed value was used for imputation; for each variable, < 1% of values were missing and, therefore, imputed this way. Means of annual estimates for each neighborhood variable were computed across child ages 0–7 years (1991–1998). The mean median family income variable was reverse scored and all five neighborhood variables were standardized, summed, and re-standardized to form a single composite of childhood neighborhood-level socioeconomic disadvantage, reflecting objective, census-derived data, with higher scores reflecting greater disadvantage.

#### Child neighborhood disadvantage (subjective assessment)

In the SECCYD follow-up study, SHINE participants completed a self-report questionnaire pertaining to the socioeconomic conditions of their childhood neighborhoods. Questions included a subset of items from The Places You’ve Lived Interview [22, 23], modified with respect to the referenced timeframe and the use of 5-point Likert response scales as follows: Participants were asked to think about the neighborhood where they lived for the longest amount of time growing up (before age 16) and to rate the veracity (1 = not at all true…5 = completely true) or frequency (1 = never…to 5 = all the time) for each item as indicated. Items described (1) automobile traffic on their street (i.e., a steady stream of cars); (2) the condition of their street (i.e., many sizable cracks, potholes, or broken curbs); (3) noise on their street (i.e., difficult to hear a person talking near to me); (4) graffiti on buildings, signs, or walls; (5) litter on their street, yard, or alley (e.g., garbage, broken glass, bottles, cigarette packages or butts, or drug related paraphernalia); (6) violent acts on their street (e.g., fist fights, beatings or use of weapons such as knives or guns); and (7) people using drugs or drinking alcohol on their street. The mean of responses was computed to form a single composite of childhood neighborhood-level socioeconomic disadvantage, reflecting participants’ subjective, retrospective reports, with higher scores reflecting greater disadvantage. In the current sample, internal consistency reliability for the neighborhood socioeconomic disadvantage scale was high (α = 0.82).

#### Negative family climate

In the SECCYD follow-up study, SHINE participants completed the relationship subscales of the Family Environment Scale (FES) [24], assessing their perceptions of their family life during childhood. On a 5-point scale, response choices indicated the level of agreement (1 = strongly disagree… 5 = strongly agree) with each statement [25]. Items were then summed to produce scores for each relationship subscale. Next, the mean of the Family Conflict subscale and the Family Cohesion subscale (reverse scored) was computed to form a composite indexing negative family climate in childhood, with higher scores reflecting greater family dysfunction. Internal consistency (α = 0.61–0.78) and test–retest (*r* = 0.52–0.91) reliabilities for the FES are adequate and validity of the FES is supported by studies showing the FES to distinguish distressed from non-distressed families [24]. In the current sample, internal consistency reliabilities for the Family Conflict (α = 0.86) and the Family Cohesion (α = 0.87) subscales were both high.

### Outcomes: positive change in response to the COVID-19 pandemic

Following participation in the SHINE study, participants additionally completed the Epidemic-Pandemic Impacts Inventory (EPII) [26]. The EPII was selected for administration following review of measures available through the NIH Repository of COVID-19 Research Tools on the US Department of Health and Human Services, NIH Public Health Emergency and Disaster Research Response (DR2) website: https://dr2.nlm.nih.gov/. The EPII was designed to assess impacts of the COVID-19 pandemic on a variety of domains related to personal and family life. Subjects were asked: “Since the coronavirus disease pandemic began, what has changed for you and your family”? Subjects then responded to 92 specific statements or events by indicating “yes” or “no” regarding whether (1) they were impacted by the event themselves; (2) another person in the home was impacted by the event; or (3) the event was not relevant to them. In addition, in alignment with other life events questionnaires, a modification was made to the original questionnaire in which all events endorsed “yes” were rated using 5 ordinal categories reflecting the intensity of the impact: 0 = no impact…2 = moderate negative/positive impact…4 = extreme negative/positive impact, according to the valence of the event. Because the EPII is newly developed, research supporting its scoring and psychometric properties is currently limited. Use of the EPII in the current study will contribute to the knowledge base regarding the further development and potential refinement of this questionnaire.

The current study focused on the subset of questions on the EPII pertaining to positive changes or events that resulted from the COVID-19 pandemic. An overall score reflecting the total number of positive events was examined as well as subscale scores representing positive changes on three dimensions: (1) *perspective-taking and charitable giving* (i.e., more appreciative of things usually taken for granted; volunteered time to help people in need; donated time or goods to a cause related to the disease; and found greater meaning in work, employment, or school); (2) *social relationships* (i.e., more quality time with family or friends in person or from a distance; improved relationships with family or friends; and new connections made with supportive people); and (3) *health behaviors* (i.e., increase in exercise or physical activity; paid more attention to personal health; ate healthier foods; used less alcohol or substances; and spent less time on screens or devices outside of work hours). Specifically, items that were endorsed “yes” (in reference to the respondent) *and* rated 1+ on the impact rating scale (0 = no impact… 2 = moderate positive impact… 4 = extreme positive impact) were summed to produce a (1) ‘positive change total’ score (12 items, 0–12); (2) ‘positive change in perspective-taking and charitable giving’ subscale score (4 items, range 0–4); (3) ‘positive change in social relationships’ subscale score (3 items, range 0–3); and (4) ‘positive change in health behaviors’ subscale score (5 items, range 0–5). The total and subscale scores were limited to items with impact ratings of 1+ to ensure that the indicated positive changes were ones that had a meaningful impact on the participants’ lives.

### Analytical plan

Separate linear regression models were fit to examine early life factors in relation to self-reported positive change events (calculated as the simple sum of endorsed items rated 1+ on impact rating scale), occurring in response to the COVID-19 pandemic. In stepwise analyses, sociodemographic variables, including gender, race/ethnicity, child SES (i.e., composite of parental education and family income-to-needs ratio) and adult SES (i.e., composite of individual education and household income) were entered on step 1. Next, early life factors, including negative family climate in childhood and neighborhood disadvantage in childhood were entered on step 2. Separate models were fit examining neighborhood disadvantage assessed objectively (i.e., composite of neighborhood characteristics in childhood derived using US Census data) versus neighborhood disadvantage assessed subjectively (i.e., composite of retrospective perceptions of neighborhood conditions in childhood). In addition, all models were fit to each of the 4 dependent variables: (1) ‘positive change total’ score; (2) ‘positive change in perspective-taking and charitable giving’ subscale score; (3) ‘positive change in social relationships’ subscale score, and (4) ‘positive change in health behaviors’ subscale score. Details pertaining to the way in which each variable was derived, coded, and composited (as relevant) are provided in the Measures section. Only one participant had missing values (i.e., no residential address information) for the variables pertaining to neighborhood disadvantage in childhood (assessed objectively). For analyses involving this variable, the sample *n* was 373 (vs. 374).

Results are reported from the final, adjusted models with all specified predictors examined simultaneously. In addition, change statistics are reported for each step of the two-step models, reflecting the contribution of the sociodemographic factors and the additional contribution of the early life factors in predicting each of the positive change outcomes. Linear regression assumptions were evaluated by visual inspection as well as by diagnostic analyses revealing general conformity to guidelines (e.g., normality of residuals by inspection of predicted probability plots, homoscedasticity by inspection of plots of predicted values and residuals, and multicollinearity by correlation coefficients and variance inflation factor values). Nevertheless, an alternative analytical approach was employed in which Poisson regression models were fit to examine the dependent variables as “counts” of experiences or events self-reported by participants since the COVID-19 pandemic began. Results of these analyses were identical to those found using linear regression models. Therefore, the linear regression models were retained for presentation. Analyses were performed in October 2020 using Stata 13 statistical software.

## Results

### Descriptive analyses

In Table 1, the sample characteristics are reported regarding sociodemographic information, early life factors, and positive change outcomes. The sample (57% female) on average was 29.1 (0.23) years old at the time the positive change outcomes were assessed. The racial/ethnic composition was 79.7% white, non-Hispanic (NH), 6.7% Latino, 8.8% black NH, 1.6% Asian NH, and 3.2% ‘other’ NH. With respect to child SES, 45.9% of mothers and 42.5% of fathers/partners received a college degree or greater by the child’s age 1 month and the average family income was more than 3 times the poverty line in the period between the child’s birth and grade 1. With respect to adult SES, 62.8% of individuals received a college degree or greater and 24.2% reported a household income of $100,000 or greater. Inspection of participants’ neighborhoods in childhood (derived as the means of annual estimates between child ages 0–7) showed the average percent of residents without a HS diploma or GED was 15.6%, ranging between 1.9% and 76.0%; the average median family income (indexed to 2010 dollars) was $76,419, ranging between $26,359 and $186,678; the average percent of men who were unemployed or out of the work force was 4.8%, ranging between 0.6% and 26.4%, the average percent of families receiving public assistance was 3.9%, ranging between 0.2% and 23.2%; and the average percent of families living below the poverty threshold was 8.8%, ranging between 0.6 and 41.0%. Finally, the individual items represented in the positive change total and subscale scores, showed items regarding perspective-taking and charitable giving ranged in frequency between 7.8% reporting having ‘volunteered to help people in need’ to 70.6% reporting feeling ‘more appreciative of things’. Items regarding social relationships ranged in frequency between 18.4% reporting having made ‘new connections with supportive people’ to 65.0% reporting experiencing ‘more quality time with family or friends’. Items regarding health behaviors ranged between 6.1% reporting ‘spending less time on screens or devices outside of work’ to 44.9% reporting having ‘paid more attention to personal health’.

**Table 1:** Sample characteristics pertaining to sociodemographic information and early life factors, including family climate and neighborhood SES in childhood, as well as positive change outcomes (*n* = 374)

	*n* (%)	Mean (SD)	Range
**Sociodemographic information:**^†^			
Age (years)	–	29.1 (0.2)	28.6–29.5
Gender (% female)	213 (57.0)	–	–
*Race/ethnicity*			
White, NH (%)	298 (79.7)	–	–
Non–white (%):	76 (20.3)	–	–
Latino (%)	25 (6.7)	–	–
Black, NH (%)	33 (8.8)	–	–
Asian, NH (%)	6 (1.6)	–	–
Other, NH (%)	12 (3.2)	–	–
*Child SES*			
Mother, % college degree +	172 (45.9)	–	–
Father/partner, % college degree +	159 (42.5)	–	–
Income-to-needs ratio	–	3.6 (2.3)	0.23–13.8
*Adult SES*:			
Individual, % college degree +	235 (62.8)	–	–
Household income, % $100,000/year +	90 (24.1)	–	–
**Early life factors**^‡^			
*Child family climate*			
Family cohesion	–	33.8 (6.4)	(9.0–45.0)
Family conflict	–	31.7 (7.0)	(9.0–45.0)
*Child neighborhood*			
Education, % without HS diploma or GED	–	15.6 (11.2)	1.9–76.0
Income, median family income	–	$76,419 (25, 958)	$26,359–$186,678
Employment, % unemployed or out of work force	–	4.8 (3.1)	0.6–26.4
Public assistance, % on public assistance	–	3.9 (3.6)	0.2–23.2
Poverty, % below poverty line	–	8.8 (7.3)	0.6–41.0
Perceptions of neighborhood, self-reported	–	9.7 (3.7)	(6.0–29.0)
**Positive change outcomes:**^¶^			
*Positive change total score*	–	3.6 (2.4)	0–12
*Perspective-taking/charitable giving subscale score*	–	1.2 (0.9)	0–4
More appreciative of things usually taken for granted (%)	264 (70.6)	–	–
Volunteered to help people in need (%)	29 (7.8)	–	–
Donated time or goods to cause related to the disease (%)	52 (13.9)	–	–
Found greater meaning in work, employment, or school (%)	99 (26.5)	–	–
*Social relationships subscale score*	–	1.2 (1.0)	0–3
More quality time with family or friends (%)	243 (65.0%)	–	–
Improved relationships with family or friends (%)	152 (40.6%)	–	–
New connections made with supportive people (%)	69 (18.4%)	–	–
*Health behaviors subscale score*	–	1.2 (1.2)	0–5
Increased exercise or physical activity (%)	101 (27.0%)	–	–
Paid more attention to personal health (%)	168 (44.9%)	–	–
Ate healthier foods (%)	117 (31.3%)	–	–
Used less alcohol or substances (%)	48 (12.8%)	–	–
Spent less time on screens or devices outside of work (%)	23 (6.1%)	–	–

^†^Age is reported from the time of completion of the EPII. Parental education is reported from child’s age 1 month. Income-to-needs ratio is reported as the mean of 8 assessments at timepoints (child’s ages 1, 6, 15, 24, 36, and 54 months and in kindergarten and grade 1)

^‡^Family climate dimensions are derived from the Family Environment Scale (FES). Child neighborhood is reported as the mean of annual estimates of socioeconomic indicators between child’s ages 0–7 years. Perceptions of neighborhood is reported from retrospective reports in adulthood using items adapted from the Neighborhood Disruption Scale (NDS)

^¶^Positive change outcomes include individual items endorsed “yes” that are also rated 1+ on the impact rating scale (0 = no positive impact…2 = moderate positive impact…4 = extreme positive impact); means (SDs) of impact ratings are reported for these items

*NH* non-Hispanic, *SES* socioeconomic status, *HS*  high school, *GED* general education diploma

### Unadjusted analyses

In Table 2, correlations are reported reflecting unadjusted associations among the sociodemographic variables, early life factors, and the positive change outcomes. Patterns emerged showing white, non-Hispanic (vs. minority) race/ethnicity was significantly associated with higher child and adult SES, as well as lower neighborhood disadvantage in childhood, indexed by US Census-derived indicators of SES (all *p*s < 0.001). As expected, child SES and adult SES were significantly correlated (*r* = 0.53, *p* < 0.001) and higher child and adult SES were inversely related to neighborhood disadvantage in childhood, according to US Census data as well as subjective reports of neighborhood conditions in childhood (all *p*s < 0.001). Negative family climate, a composite of low family cohesiveness and high family conflict was not significantly related to any of the sociodemographic, neighborhood, or positive change outcome variables (all *p*s > 0.05). Examination of the sociodemographic and neighborhood variables in relation to the positive change outcomes, showed white, non-Hispanic (vs. minority) race/ethnicity was significantly related to a lower number of positive change events on the ‘positive change total’ scale and the ‘positive change in health behaviors’ subscale (*p*s < 0.05). In addition, a pattern emerged showing greater neighborhood disadvantage in childhood, according to objective and subjective assessments, was related to a higher number of positive change events on the ‘positive change total’ scale (*p*s between < 0.05 and < 0.01), the ‘positive change in perspective-taking and charitable giving’ subscale (*p*s < 0.05), and the ‘positive change in social relationships’ subscale (*p*s between < 0.05 and < 0.01).

**Table 2:** Bivariate correlations among the sociodemographic, early life, and positive change outcome variables (*n* = 374)

Variables:^†^	1	2	3	4	5	6	7	8	9	10	11	12	13	14	15
1. Gender	–	.017	.023	− .041	− .092	− .035	.005	− .068	− .043	− .034	− .111*	.047	.042	.053	.016
2. Race		–	.241***	.200***	.044	− .296***	.247***	− .376***	− .385***	− .364***	− .083	− .111*	− .035	− .099†	− .107*
3. Child SES			–	.526***	.061	− .461***	.581***	− .339***	− .389***	− .337***	− .287***	.035	.079	− .019	.023
4. Adult SES				–	− .013	− .268***	.350***	− .281***	− .372***	− .298***	− .312***	.047	.068	.033	.014
5. Neg family climate					–	.002	.015	.024	.072	.006	.001	− .074	− .091	− .063	− .024
6. NEIGH− low education						–	− .652***	.511***	.628***	.511***	.347***	.109*	.093†	.141**	.028
7. NEIGH− income							–	− .506***	− .581***	− .638***	− .335***	− .022	− .044	− .041	.023
8. NEIGH-unemployment								–	.803***	.729***	.390***	.128*	.085	.164**	.051
9. NEIGH-public assist									–	.763***	.366***	.128*	.068	.119*	.100†
10. NEIGH-poverty										–	.319***	.143**	.114*	.147**	.071
11. NEIGH-subjective											–	.110*	.113*	.100†	.047
12. POS-total												–	.666***	.762***	.810***
13. POS-perspective													–	.322***	.278***
14. POS-social														–	.422***
15. POS-health															–

^†^Gender was coded (0 = male, 1 = female); Race/ethnicity was coded (0 = non-white, 1 = white non-Hispanic); Child SES and Adult SES were coded with higher values reflecting higher SES; Neg family climate was coded with a higher value reflecting higher family dysfunction; NEIGH-low education, NEIGH-unemployment, NEIGH-public asst, NEIGH-poverty, and NEIGH-subjective were coded with higher values reflecting greater neighborhood disadvantage; NEIGH-income was coded with a higher value reflecting greater median family income; POS variables were coded with higher values reflecting a higher number of positive change events

### Adjusted analyses

**Fig. 1:**
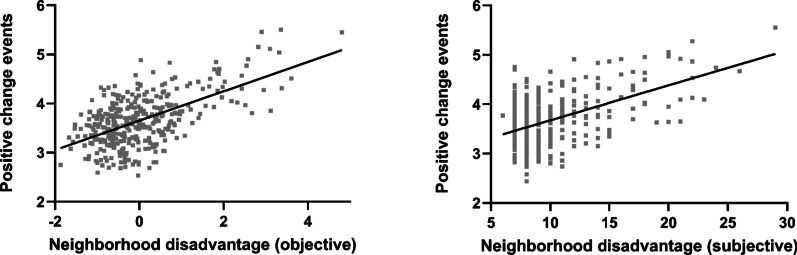
Scatterplots depict the adjusted, significant associations between child neighborhood disadvantage, measured both objectively and subjectively, and the ‘positive change total’ score

In Table 3, results of the final, adjusted models are reported for effects of the sociodemographic variables and early life factors (negative family climate and neighborhood disadvantage) on each positive change outcome: (1) ‘positive change total’ score; (2) ‘positive change in perspective-taking and charitable giving’ subscale score; (3) ‘positive change in social relationships’ subscale score, and (4) ‘positive change in health behaviors’ subscale score. With respect to the early life factors, in the final, adjusted models, child neighborhood disadvantage, assessed objectively using US Census data, predicted the ‘positive change total’ score (Model 1a: *β* = 0.18, *p* = 0.004). That is, living in a more disadvantaged neighborhood as a child predicted a higher number of positive change events experienced as an adult in response to the COVID-19 pandemic. This association was independent of sociodemographic factors, including gender, race, child SES, and adult SES, as well as negative family climate in childhood. The model change statistics showed the sociodemographic variables accounted for 2.1% of the variance and the early life factors accounted for an additional 2.6% of the variance in the ‘positive change total’ score. In parallel, greater child neighborhood disadvantage, assessed by subjective reports of participants’ perceptions of their childhood neighborhoods, also predicted the ‘positive change total’ score (Model 1b: *β* = 0.15, *p* = 0.006) and the model change statistics showed the early life factors accounted for an additional 2.4% of the variance in the ‘positive change total’ score. In Fig. 1, scatterplots depict the adjusted, significant associations between child neighborhood disadvantage, measured both objectively and subjectively, and the ‘positive change total’ score.

**Table 3:** Regression analyses examining early life factors in relation to positive change outcomes, including the positive change total score and subscales: perspective-taking and charitable giving, social relationships, and health behaviors

	*b*	95% CI	*β*	sig	Model change statistics for each step			
					R^2^	R^2^_change_	F	F sig
**DV: Positive change total**								
*Model 1a*								
Step 1: Gender	0.243	(− 0.241, 0.727)	.051	.324	.021	−	1.975	.098
Race/ethnicity	− 0.455	(− 1.101, 0.191)	− .077	.167				
Child SES	0.260	(− 0.046, 0.565)	.109	.095				
Adult SES	0.176	(− 0.108, 0.461)	.074	.223				
Step 2: Child negative family climate	− 0.066	(− 0.153, 0.022)	− .076	.143	.047	.026	4.968	.007
Child neighborhood disadvantage (objective)	0.432	(0.137, 0.727)	.182	.004				
*Model 1b*:								
Step 1: Gender	0.298	(− 0.189, 0.785)	.062	.230	.021	−	1.979	.097
Race/ethnicity	− 0.766	(− 1.377, − 0.155)	− .130	.014				
Child SES	0.156	(− 0.131, 0.444)	.066	.286				
Adult SES	0.209	(− 0.079, 0.497)	.088	.154				
Step 2: Child negative family climate	− 0.056	(− 0.144, 0.031)	− .065	.205	.045	.024	4.705	.010
Child neighborhood disadvantage (subjective)	0.098	(0.029, 0.168)	.153	.006				
**DV: Positive change in perspective-taking and charitable giving**								
*Model 2a*:								
Step 1: Gender	0.077	(− 0.109, 0.264)	.042	.415	.013	−	1.182	.318
Race/ethnicity	− 0.001	(− 0.250, 0.248)	− .001	.992				
Child SES	0.139	(0.022, 0.257)	.152	.020				
Adult SES	0.058	(− 0.052, 0.167)	.063	.300				
Step 2: Child negative family climate	− 0.033	(− 0.067, 0.000)	− .100	.053	.047	.034	6.532	.022
Child neighborhood disadvantage (objective)	0.183	(0.069, 0.297)	.200	.022				
*Model 2b*:								
Step 1: Gender	0.101	(− 0.087, 0.288)	.055	.291	.013	−	1.177	.320
Race/ethnicity	− 0.133	(− 0.369, 0.102)	− .059	.266				
Child SES	0.096	(− 0.015, 0.206)	.105	.090				
Adult SES	0.072	(− 0.039, 0.183)	.079	.203				
Step 2: Child negative family climate	− 0.029	(− 0.063, 0.004)	− .089	.086	.045	.033	6.284	.002
Child neighborhood disadvantage (subjective)	0.042	(0.015, 0.069)	.169	.002				
**DV: Positive change in social relationships**								
*Model 3a*:								
Step 1: Gender	0.120	(− 0.081, 0.322)	.061	.242	.017	−	1.583	.178
Race/ethnicity	− 0.129	(− 0.398, 0.141)	− .053	.348				
Child SES	0.038	(− 0.089, 0.166)	.039	.554				
Adult SES	0.092	(− 0.027, 0.210)	.093	.128				
Step 2: Child negative family climate	− 0.022	(− 0.059, 0.015)	− .061	.239	.041	.024	4.669	.010
Child neighborhood disadvantage (objective)	0.181	(0.058, 0.304)	.183	.004				
*Model 3b*:								
Step 1: Gender	0.138	(− 0.066, 0.341)	.069	.185	.017	−	1.590	.176
Race/ethnicity	− 0.259	(− 0.514, − 0.003)	− .106	.047				
Child SES	− 0.009	(− 0.129, 0.111)	− .009	.885				
Adult SES	0.100	(− 0.021, 0.220)	.101	.104				
Step 2: Child negative family climate	− 0.018	(− 0.055, 0.018)	− .051	.328	.034	.017	3.199	.042
Child neighborhood disadvantage (subjective)	0.034	(0.005, 0.063)	.128	.020				
**DV: Positive change in health behaviors**								
*Model 4a*:								
Step 1: Gender	0.046	(− 0.214, 0.305)	.018	.729	.014	−	1.353	.250
Race/ethnicity	− 0.325	(− 0.671, 0.021)	− .105	.066				
Child SES	0.082	(− 0.081, 0.246)	.066	.324				
Adult SES	0.027	(− 0.126, 0.179)	.021	.731				
Step 2: Child negative family climate	− 0.010	(− 0.057, 0.037)	− .023	.667	.017	.002	.429	.651
Child neighborhood disadvantage (objective)	0.068	(− 0.090, 0.226)	.054	.398				
*Model 4b*:								
Step 1: Gender	0.059	(− 0.201, 0.320)	.024	.654	.015	−	1.358	.248
Race/ethnicity	− 0.375	(− 0.701, − 0.048)	− .121	.025				
Child SES	0.069	(− 0.084, 0.223)	.056	.375				
Adult SES	0.037	(− 0.116, 0.191)	.030	.479				
Step 2: Child negative family climate	− 0.009	(− 0.055, 0.038)	− .019	.713	.019	.004	.750	.473
Child neighborhood disadvantage (subjective)	0.022	(− 0.015, 0.059)	.065	.244				

*b* = unstandardized regression coefficient; 95% CI = 95% confidence interval for *b*; *β* = standardized regression coefficient; R^2^ = R-squared or variance explained for the step; R^2^_change_ = change in R^2^ or variance explained for the step

The three subscales that comprise the ‘positive change total’ score were then examined as outcomes in separate models. In these analyses, reported from the final, adjusted models, greater child neighborhood disadvantage predicted the ‘positive change in perspective-taking and charitable giving’ subscale score, according to both objective (Model 2a: *β* = 0.20, *p* = 0.022) and subjective (Model 2b: *β* = 0.17, *p* = 0.002) assessments of child neighborhood. Model change statistics showed the sociodemographic variables accounted for 1.3% and the early life factors accounted for an additional 3.4% of the variance in ‘positive change in perspective-taking and charitable giving’ subscale. In parallel, greater child neighborhood disadvantage also predicted the ‘positive change in social relationships’ subscale score, according to both objective (Model 3a: *β* = 0.18, *p* = 0.004) and subjective (Model 3b: *β* = 0.13, *p* = 0.020) assessments of child neighborhood. The model change statistics showed the sociodemographic variables accounted for 1.7% and the early life factors accounted for an additional 2.4% of the variance in ‘positive change in social relationships’ subscale. In contrast, early life factors were unrelated to the ‘positive change in health behaviors’ subscale score (*p*s > 0.05).

With respect to the sociodemographic variables, reported from the final, adjusted models, minority (vs. white) race/ethnicity was significantly associated with a higher number of positive change events with respect to outcomes: ‘positive change total’ score (Model 1b: *p* = 0.014), ‘positive change in social relationships’ subscale score (Model 3b: *p* = 0.047), and ‘positive change in health behaviors subscale score (Model 4b: *p* = 0.025). In addition, higher child SES was significantly associated with the ‘positive change in perspective-taking and charitable giving’ subscale score (Model 2a: *p* = 0.020).

## Discussion

The goal of the current study was to examine positive changes experienced by individuals in response to the COVID-19 pandemic and to determine whether the capacity of individuals to enact such changes may be shaped by prior adversities experienced in childhood. Results showed that individuals who grew up in more disadvantaged neighborhoods, indexed both by objective and subjective assessments of neighborhood conditions, reported more positive change events in response to the pandemic. Associations were independent of key sociodemographic factors and childhood family dysfunction. These findings are broadly consistent with prior studies of resilience and stress-related growth that show some individuals exhibit positive changes in response to stressful life events [14–16]. Going a step further, these findings also suggest that the capacity to enact such positive changes may be rooted in early life experiences. Specifically, the “hidden talents” model posits that adaptations to stress in childhood may facilitate stress-adapted skill sets which help individuals to negotiate and possibly better cope with future threats [21]. Findings from the current study warrant additional investigation to better understand how disadvantage experienced in early life may shape prosocial responses to stress in adulthood.

When the subscales of the ‘positive change total’ were examined separately, it revealed that individuals who lived in more disadvantaged neighborhoods as children reported more positive change events in areas of ‘perspective taking and charitable giving’ and ‘social relationships’, but not ‘health behaviors.’ ‘Perspective taking and charitable giving’ entailed items reflecting greater feelings of appreciation, finding greater meaning in work or school, and donating time or goods to help others. ‘Social relationships’ entailed items reflecting more quality time and improved relationships with family or friends and new connections made with supportive people. These findings showing increases in generosity and social connections are partially consistent with prior studies in which individuals from disadvantaged backgrounds exhibited more sensitivity to social contexts and more attunement to the needs of others [27, 28]. In a series of studies, including one in which social status was manipulated, individuals of lower social standing consistently exhibited more prosocial behaviors, reflecting greater generosity, charitability, and help-giving than their higher status counterparts [29]. Moreover, in a large, nationally representative sample, lower SES, indexed by household income, was associated with other-focused emotions (i.e., compassion, love, awe) while higher SES with associated with self-focused emotions (i.e., contentment, pride, amusement) [30].

Findings from the current study, however, differ from the studies mentioned above [27–30] in two important ways. First, the current findings pertain to neighborhood (vs. individual) SES. In fact, greater neighborhood disadvantage related to more positive change events, independently of individual child and adult SES; and patterns of association between individual child and adult SES and number of positive change events were actually positive in their direction of association, albeit non-significant. Second, the current findings pertain to adversity experienced in childhood specifically. The integration of this work suggests that growing up in harsh conditions typical of low SES neighborhoods (i.e., high threat, high unpredictability, and low control) may promote stress-related adaptations that shape the prosocial emotional (e.g., appreciation) and behavioral (e.g., donating to charity) responses observed in the current study. It is plausible that these adaptations may aid in the protection of individuals, families, and communities when facing adversity such as the COVID-19 pandemic. In contrast, children living in middle- and upper-class neighborhoods may be insulated from such exposures, lessening the opportunity to build the same capacity for gratitude, charitability, and social connection. Finally, it is also worth noting that while participants in the current study experienced relatively high SES as children (46% of mothers had a college or graduate degree), there was substantial variability in child neighborhood SES. For example, the percent of residents without a HS diploma or GED ranged from 1.9% to 76.0%, the percent of men who were unemployed or out of the work force ranged from 0.6% to 26.4%, and the percent of families living below the poverty threshold ranged from 0.6 to 41.0%.

## Strengths and weaknesses

Strengths of the current study include its timely focus on understanding impacts of the COVID-19 pandemic on the well-being of individuals as well as its novel consideration of impacts that are positive, versus maladaptive, in nature, including increases in perspective-taking and charitable giving, as well as improvements in social relationships and health behaviors. An additional strength is its representation of a subset of subjects who have been participating in a larger, longitudinal study beginning at their birth. In this context, available data over the life course were able to be leveraged for analysis. Specifically, rigorous measurement of neighborhood disadvantage in childhood was achieved using state-of-the-art methods. Residential addresses obtained and updated continuously through early childhood (birth to 7 years) were used to derive annual estimates, by linear interpolation, of neighborhood-level SES indicators from decennial US Censuses. In addition, a second method of assessment was employed in which retrospective self-reports of neighborhood conditions in childhood were obtained. Notably, results using these two different measurement strategies converged to show the same pattern of results, further strengthening confidence in the study findings.

On the other hand, the current study has several weaknesses. The questionnaire used to assess impacts of the COVID-19 pandemic (EPII) was newly constructed with limited information available regarding its validity and reliability. In addition, assessments of relevant constructs that could have informed the study findings, such as measures of social support, coping strategies, or resiliency, were not available. As well, the mechanism—adaptations to stress—postulated to link neighborhood disadvantage in childhood to positive change events in adulthood is inferred only and not explicitly tested. Another weakness of the current study is its relatively small sample size and that this subset of participants was not representative of the larger, original sample. That is, individuals who participated in the current study, versus individuals from the original study who did not participate, experienced higher SES as children, indexed by higher parental education and higher family income-to-needs ratios. It is noteworthy that some of these weaknesses are due to the impromptu and opportunistic nature of the current study as a focus on the COVID-19 pandemic was obviously unanticipated when the original study and its follow-up were designed.

## Critical analysis and future directions

The current study drew on the resilience and stress-related growth literatures for conceptual framing. Recent critiques, however, highlight limitations of these literatures, including analytical approaches that erroneously magnify the prevalence of resilience, an overreliance on single indicators of resilience, retrospective examinations of growth, and poor correspondence between perceived growth and actual change [31–34]. These concerns, along with the weaknesses of the current study noted above, point to areas for improvement in future research investigations. Here, we integrate and extend recommendations for future research [31] in the context of findings from the current study.

First, future studies should examine outcomes on multiple dimensions as individuals’ responses to adversity may vary across dimensions. In the current study, it is notable that neighborhood disadvantage in childhood predicted positive change events in response to the COVID-19 pandemic in areas of ‘perspective taking and charitable giving’ and ‘social relationships’, but not ‘health behaviors.’ Differential effects on these positive change events may reflect the activation of emotions and behaviors that are helpful in coping with the COVID-19 pandemic (e.g., greater feelings of appreciation, volunteering to help others, improved relationships) versus behaviors that are more limited to the benefit one’s personal well-being (e.g., increases in exercise and healthy eating). Second, future studies should be longitudinal in design, including the prospective examination of mechanisms that may explain variability in responses to adversity. In the current study, we described stress-related adaptations or “hidden talents” [21] as potential mechanisms linking neighborhood disadvantage in childhood to adulthood responses to the COVID-19 pandemic but did not measure such mechanisms directly. The identification of mechanisms in the “hidden talents” framework may include real-time childhood assessments of criterion-based skills in areas, for example, of empathy and social attunement which may, in turn, promote help giving behaviors and positive social relationships when facing adversity in adulthood. Third, future studies should measure the outcomes of interest before and after the adversity exposure to assess actual change. In the current study, participants were asked: “since the coronavirus disease pandemic began, what has changed for you and your family”. Research, however, suggests there may be poor correspondence between perceived change and measured change and that individuals have difficulty both in judging pre-adversity levels of functioning and in making appropriate attributions for the reported perceived change [33, 34]. Finally, future studies should consider the nature of the adversity exposures of interest. Clearly, there are numerous adversity exposures and individuals’ responses may vary depending on the characteristics of the exposure. The adversity of neighborhood disadvantage, for example, may result in a unique set of adaptations that do not necessarily emerge in the context of other types of adversity. Likewise, the positive change events examined in response to the COVID-19 pandemic may not generalize in response to other stressors.

## Conclusions

Results of the current study revealed that individuals who grew up in more disadvantaged neighborhoods, indexed both by objective and subjective assessments of neighborhood conditions, reported more positive change events in response to the COVID-19 pandemic, a stressful life event. Associations were independent of key sociodemographic factors and childhood family dysfunction. Findings extend our current knowledge of early life adversity by considering the developmental context of positive, versus maladaptive, outcomes. Among the several research recommendations described above, additional research is needed to delineate how adversity may shape prosocial responses to stress and the specific adaptations to stress that may mediate these associations. Leveraging strengths, versus deficits, that emerge in response to early life adversity may inform intervention efforts to reduce well-established negative impacts of early life adversity on a myriad of poor mental and physical health outcomes.

## Data Availability

Data from the original NICHD SECCYD are available on this website: icpsr.umich.edu/web/ICPSR/series/233. Data and materials from the SECCYD follow-up study, SHINE, are in progress and have not been made available on a permanent third-party archive; requests regarding access to the data and materials can be sent via email to the lead author at mbleil@uw.edu.

## Abbreviations

COVID-19: Coronavirus disease 2019
EPI: Epidemic-Pandemic Impacts Inventory
FES: Family Environment Scale
SECCYD: Study of Early Child Care and Youth Development
SES: Socioeconomic status
SHINE: Study of Health in Early and Adult Life
